# Orthologous genes identified by transcriptome sequencing in the spider genus *Stegodyphus*

**DOI:** 10.1186/1471-2164-13-70

**Published:** 2012-02-14

**Authors:** Tiina M Mattila, Jesper S Bechsgaard, Troels T Hansen, Mikkel H Schierup, Trine Bilde

**Affiliations:** 1 Department of Biology, University of Oulu, PO Box 3000, FIN-90014 Oulu, Finland; 2 Department of Bioscience, Aarhus University, Ny Munkegade 116, Building 1540, DK-8000 Aarhus C, Denmark; 3 Bioinformatics Research Centre, Aarhus University, C. F. Møllers Alle, DK-8000 Aarhus C, Denmark

## Abstract

**Background:**

The evolution of sociality in spiders involves a transition from an outcrossing to a highly inbreeding mating system, a shift to a female biased sex ratio, and an increase in the reproductive skew among individuals. Taken together, these features are expected to result in a strong reduction in the effective population size. Such a decline in effective population size is expected to affect population genetic and molecular evolutionary processes, resulting in reduced genetic diversity and relaxed selective constraint across the genome. In the genus *Stegodyphus*, permanent sociality and regular inbreeding has evolved independently three times from periodic-social (outcrossing) ancestors. This genus is therefore an ideal model for comparative studies of the molecular evolutionary and population genetic consequences of the transition to a regularly inbreeding mating system. However, no genetic resources are available for this genus.

**Results:**

We present the analysis of high throughput transcriptome sequencing of three *Stegodyphus *species. Two of these are periodic-social (*Stegodyphus lineatus *and *S.tentoriicola*) and one is permanently social (*S. mimosarum*). From non-normalized cDNA libraries, we obtained on average 7,000 putative uni-genes for each species. Three-way orthology, as predicted from reciprocal BLAST, identified 1,792 genes that could be used for cross-species comparison. Open reading frames (ORFs) could be deduced from 1,345 of the three-way alignments. Preliminary molecular analyses suggest a five- to ten-fold reduction in heterozygosity in the social *S. mimosarum *compared with the periodic-social species. Furthermore, an increased ratio of non-synonymous to synonymous polymorphisms in the social species indicated relaxed efficiency of selection. However, there was no sign of relaxed selection on the phylogenetic branch leading to *S. mimosarum*.

**Conclusions:**

The 1,792 three-way ortholog genes identified in this study provide a unique resource for comparative studies of the eco-genomics, population genetics and molecular evolution of repeated evolution of inbreeding sociality within the *Stegodyphus *genu*s*. Preliminary analyses support theoretical expectations of depleted heterozygosity and relaxed selection in the social inbreeding species. Relaxed selection could not be detected in the *S. mimosarum *lineage, suggesting that there has been a recent transition to sociality in this species.

## Background

Sociality in spiders involves the formation of permanent family groups and extensive cooperation in prey capture and brood care. While sociality is relatively rare in spiders with less than 25 known social species, it is intriguing because it has evolved independently at least 18 times in six families across the spider phylogeny [1]. The evolution of sociality involves convergent life history traits that include female-biased sex ratios, an inbreeding mating system, and cooperative breeding with allomaternal care [1]. The genus *Stegodyphus *contains three social species [2], and although the phylogenetic relationship of *Stegodyphus *is poorly resolved, a partial molecular phylogeny suggests three independent origins of sociality, and that sociality is a derived state [3]. Sociality is hypothesized to evolve via the subsocial route through the elimination of pre-mating dispersal, the transition to intra-colony mating, and the evolution of cooperation among individuals. The presence of independent social lineages and periodic-social (ancestral) congeners makes *Stegodyphus *a suitable system for comparative studies of the evolution of regular inbreeding and sociality, and for examining its consequences for evolutionary processes and genomic architecture.

The combination of strong inbreeding, female-biased sex ratios, and dynamic meta-population structure in the social spiders is expected to have pronounced effects on genetic processes [4,5]. Comparative data from studies on selfing and outcrossing plants are consistent with theoretical predictions that inbreeding populations are expected to experience reduced genetic diversity. This prediction is founded on three factors: firstly, that inbreeding reduces effective population size relative to outbreeding populations; secondly, high homozygosity reduces the effective frequency of recombination throughout the genome; and thirdly, isolation between populations is increased with inbreeding [4]. As a consequence, the efficacy of natural selection should be reduced. Examining these expectations and additional genomic consequences of the transition to sociality and regular inbreeding has so far not been possible due to a lack of genetic resources. Next generation sequencing technologies have made it possible to survey genomes of non-model organisms [e.g. [6-11]], and to identify suitable parts of the genome for phylogenetic, molecular evolutionary and population genetic studies. Using 454 pyrosequencing (Roche), we sequenced the transcriptomes of three *Stegodyphus *species to identify ortholog gene sequences across species to use in phylogenetic and population genetic studies.

The three *Stegodyphus *species sequenced here (*S. lineatus, S. tentoriicola and S. mimosarum*) were specifically selected to be as distantly related as possible, based on the most recent molecular phylogeny of the genus [3], to maximize the chance of finding regions conserved across the entire genus for the design of primers that will amplify in all or most *Stegodyphus *species. One social species and two periodic-social species [3] were included to perform preliminary contrasts of evolutionary patterns of the transcriptomes for inbreeding and outcrossing mating systems to substantiate evolutionary hypotheses to test in future studies.

We obtained a library of 1,792 ortholog loci and detected open reading frames (ORFs) (> 189 bp) in 1,345 loci. Preliminary analysis on the transcriptome sequence data showed that heterozygosity was strongly depleted in the social individual compared with the two periodic-social individuals (5 to 10-fold). Estimates of dN/dS, CG-content and codon usage bias did not reveal differences in the strength of selection averaged over the phylogenetic branches. However, levels of non-synonymous and synonymous polymorphisms suggest that selection is relaxed in the social *S. mimosarum *compared to the periodic-social species. From these results we hypothesize that permanent sociality evolved recently in *S. mimosarum*.

## Results and Discussion

### Assembly and characterization of transcriptomes

Three transcriptomes were assembled from quality filtered sequences into 10,398, 6,882 and 7,195 transcripts, with a median length of approximately 700 bp and median coverage of 4.2, 4.7 and 4.7 for *S. lineatus, S. mimosarum *and *S. tentoriicola *respectively (see Table 1 for original and assembled data). Based on transcript clustering, 8,944, 5,893 and 6,094 individual putative genes for each of the respective species were identified, of which approximately 8.5% were found to have more than one splice variant. The statistics for the original and assembled data are summarised in Table 1.

**Table 1:** Summary statistics of original and assembled data from transcriptomes of three *Stegodyphus *spp

	*S lineatus*	*S. mimosarum*	*S. tentoriicola*
total number of reads	457,675	542,510	459,994
mean length	361	323	342
median length	417	348	374
number of reads in assembly	331,109	234,844	366,670
number of transcripts	10,398	6,882	7,195
mean transcript length	951	920	934
median transcript length	714	716	733
mean coverage	13.1	17.7	19.6
median coverage	4.2	4.7	4.7
number of putative genes isogroups	8,944	5,893	6,094
percentage of putative genes with splice variants	8.1%	8.5%	8.4%
number of detected SNPs	6,425	943	5,043
number of detected indels	112	15	54
number of sites for SNP search	2,508,128	2,050,168	1,929,232
number of putative genes with protein blast hits in non-redundant protein database	4,296	3,535	3,766
number of putative genes with protein blast hits in *Drosophila *UniProtKB	1,077	918	993
Number of putative genes with GO annotation	3,877	3,272	3,457

**Figure 1:**
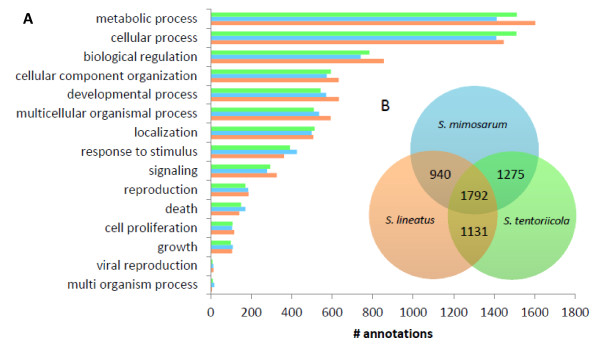
**A) Distribution of biological function annotation of three *Stegodyphus *transcriptomes**. Blue bars: *S. mimosarum*, orange bars: *S. lineatus*, and green bars: *S. tentoriicola *B) Venn diagram showing the number of detected ortholog sequences between species.

Approximately half of the putative genes in *S. lineatus *and 60% in *S. mimosarum *and *S. tentoriicola *were annotated by comparative analysis using BLASTx against the NCBI non-redundant protein database (see Table 1). Functional annotation (Gene Ontology) was similar across all three species with the highest number of annotated transcripts related to metabolic processes (GO:0008152) and cellular processes (GO:0009987) (Figure 1A). Many of these genes are probably highly conserved genes, such as genes involved in DNA repair, gene expression regulation and transfer of chemical substances. This category of genes contains the largest amount of products in the Gene Ontology (GO) database, and the abundance of transcripts in different categories in the present data set is similar to the abundance of the number of products in the GO database.

**Figure 2:**
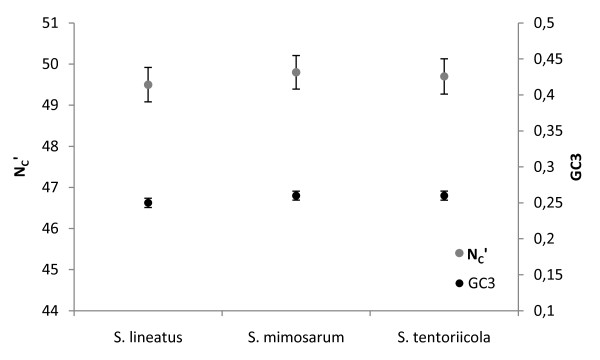
**Codon usage bias and GC content in third codon position estimates and 95% confidence intervals for three *Stegodyphus *species**. Estimates are based on concatenated genes.

The study species were found to have highly similar patterns of coding GC content and codon usage. The overall GC content of the transcriptomes was 32.2%, 34.1% and 33.2% in *S. lineatus, S. mimosarum *and *S. tentoriicola*, respectively, while in defined coding regions (see below) the GC content was slightly higher (approximately 40% in all three species) compared to the overall GC content. The patterns of codon usage in these genes, studied as the effective number of codons (N_c_') and the GC content in synonymous sites (GC_3_), were highly similar in all study species. The effective numbers of codons was approximately 50, and AT-rich codons were preferred (Figure 2). The amount of codon usage bias was of the same order of magnitude as several Diptera and Hymenoptera species [12,13]. However, while in many Diptera species the preferred codons are GC-rich, AT-rich codons were more common in *Stegodyphus*. Thirty-seven Nematode species have shown large variation in GC content of preferred codons, but, with a few exceptions, the GC content in third codon position is generally much higher in Nematode genomes than found in the spider species here [14].

### Orthology

1,792 transcripts were found to have reciprocal best hit in all three pair-wise comparisons, and were considered to be ortholog across all three study species. However, 940, 1,131 and 1,275 respective sequences were found to have best reciprocal hits between two species only (Figure 1B.). Of these sequences, 261 *S. lineatus*, 241 *S. mimosarum *and 259 *S. tentoriicola *transcripts showed reciprocal best hits among two species pairs only, which might have been caused by technical issues or paralogy (see Additional file 1). To avoid paralog genes in the downstream analyses, these loci were excluded.

The alignments of 1,792 ortholog sequences serve as a highly useful library for further genetic studies across the *Stegodyphus *genus. Since the three species sequenced here are phylogenetically distant within the genus, the likelihood that PCR primers designed from conserved regions in the alignments will amplify throughout the genus is maximized. Indeed, the first ten PCR primer pairs designed from ortholog sequences amplified in 8 species tested across the genus. The sequences of these primer pairs can be found in Additional file 2.

### Molecular evolution

To study patterns of molecular evolution of protein coding genes we generated multiple alignments of the longest open reading frames detected from the three way ortholog sequences. This resulted in 1,345 alignments with a median length of 171 codons ranging from 63 to 1,092. These 1,345 ortholog alignments can be found in Additional file 3. Eighty-seven percent of the longest translated ORFs had at least one significant blastp hit (e-value cut-off 10^-6^) in the NCBI non-redundant database, suggesting that in the majority of genes the correct reading frame was detected.

Single genes were concatenated into 20 units with an average length of 41,825 bp to estimate genome-wide substitution patterns, and the total length of the aligned region was 836,517 bp, gaps included. Alignments were concatenated to increase reliability of branch length estimation, and we generated 20 units (and not one single unit) to be able to estimate confidence limits. The proportion of variable sites in pair-wise comparisons was 5.7% both between *S. lineatus *and *S. mimosarum *and between *S. lineatus *and *S. tentoriicola*, and 4.2%. between *S. mimosarum *and *S. tentoriicola*. The transcriptome-wide selection against amino acid changing mutations was found to be strong in all lineages, but in the *S. lineatus *lineage selection was significantly stronger than in the other two lineages. The estimates of the ratio of non-synonymous to synonymous divergence (ω) (95% confidence limits in parentheses) were 0.094 (0.088-0.099), 0.119 (0.111-0.128) and 0.11 (0.104-0.117) in *S. lineatus, S. tentoriicola*, and *S. mimosarum *lineages respectively. This is comparable with genome-wide estimates of ω in *Drosphila *[15]. We note that because we extracted RNA from the entire body and used non-normalized cDNA libraries, genes that are expressed in all tissues are overrepresented in the dataset. These genes are likely to be under strong purifying selection [16].

### Genetic polymorphism in social and periodic-social spiders

We found strikingly fewer polymorphisms in the transcriptome from the social species compared to that of periodic-social species. From the assembled transcript in coverage range 10 to 150, we detected 6,425 and 5,043 polymorphisms for *S. lineatus *and *S. tentoriicola *respectively, while only 943 polymorphisms were detected from *S. mimosarum *transcripts. If only one transcript per gene was taken into account the difference in detected polymorphisms was even higher, with 3,901 polymorphism in *S. lineatus *and 2,415 in *S. tentoriicola *respectively, while only 591 polymorphisms were detected in the *S. mimosarum *transcript. These data are in keeping with low population level genetic variation in mitochondrial loci that were previously demonstrated in two genera of social spiders [17,18]. Our data for the social *S. mimosarum *transcriptome is consistent with the prediction of reduced population level polymorphism in social species as a consequence of inbreeding. This is the goal of future comparative studies of social and periodic-social *Stegodyphus *species.

In addition, we found evidence for a higher ratio of non-synonymous polymorphisms to synonymous polymorphisms (π(a)/π(s)) in the social species compared with the outcrossing congeners. The number of polymorphisms that could be determined as synonymous and non-synonymous were 567 and 199 for *S. lineatus*, 385 and 239 for *S. tentoriicola*, and 22 and 24 for *S. mimosarum *respectively. The estimated π(a)/π(s) were 0.10 for *S. lineatus *and 0.18 for *S. tentoriicola*, and 0.32 for the social species *S. mimosarum*, indicating a relaxation of purifying selection. Lower polymorphism and higher π (a)/π(s) in the social species suggest lower effective population size and relaxed purifying selection, as expected for highly inbred species [4,19]. Contrary to these findings, the ω estimates in different branches did not show consistent significant differences between social and periodic-social species. ω is expected to increase over evolutionary time in inbreeding social species with low effective population size and relaxed purifying selection because of increased accumulation of slightly deleterious mutation [20]. However, since we did not observe an increased ω relaxation of purifying selection in *S. mimosarum *probably only occurred relatively recently. Assuming that relaxed purifying selection followed the transition to sociality, we tentatively hypothesize that sociality and regular inbreeding in *S. mimosarum *evolved relatively recently, and that the transition was not associated with speciation.

### Genetic relationships of *Stegodyphus*

**Figure 3:**
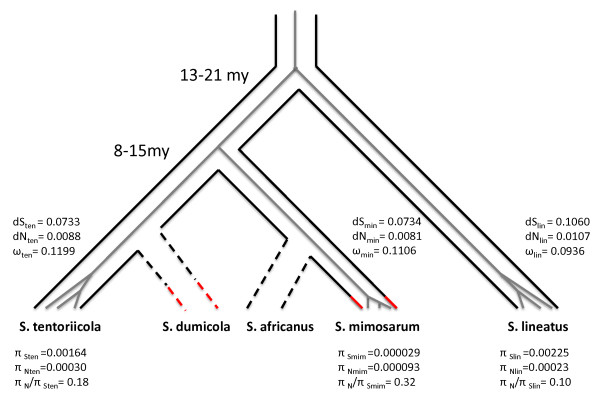
**Presumed phylogeny of three *Stegodyphus *study species**. Two species, *S. dumicola *and *S. africanus*, indicated with dashed branches, were added based on Johannesen et al (2007) to illustrate that the branches to *S. tentoriicola *and *S. mimosarum *do not represent single species only. Red branch bounds indicate social species. The splitting of the terminal branches in the species studied here represents the level of polymorphism within these three species. Split time estimates, substitution rate estimates (dN, dS and ω) and polymorphism estimates (π) were mapped on the phylogeny. The root was placed under the assumption of a molecular clock.

Mean synonymous substitution rates (dS) from concatenated genes with a free-ratio model were 0.073, (95% CI 0.072-0.075) for both *S. mimosarum *and *S. tentoriicola*, and non-synonymous substitution rates (dN) were 0.0081 (95% CI 0.0076-0.0086) and 0.0088 (95% CI 0.0082-0.0093) respectively. In the *S. lineatus *lineage, the rate estimates were 0.139 (95% CI 0.136-0.141) for dS and 0.013 (95% CI, 0.012-0.014) for dN. These data suggest that *S. mimosarum *and *S. tentoriicola *are genetically the more closely related species (Figure 3). This is supported by cytogenetic studies of chromosome numbers [21] and a recent phylogenetic study [22], but is contrary to the phylogeny estimated by Johannesen et al. [3].

Under the assumption of a molecular clock of synonymous substitutions, we estimated the time for speciation using two different types of calibration. First we estimated a 'spider mutation rate' from an estimated synonymous divergence of dS = 2.64 (1.32 on each branch) between *S. lineatus *and *Acanthoscurria gomesiana *(Theraphosidae) (based on the 16,000 bp alignment of *A. Gomesiana *haemocyte EST data [23] and data from this study). The split time between these species has been estimated at ~245 my [24,25], yielding a mutation rate of approximately 5, 4E-9 per year. *Stegodyphus *species are annual with one generation per year, therefore, based on this estimated mutation rate, we date the split between *S. mimosarum *and *S. tentoriicola *to ~15 million years ago, and the split of *S. mimosarum*/*S. tentoriicola *and *S. lineatus *to ~21 million years ago (Figure 3). We also used a direct mutation rate estimate from mutation accumulation lineages of *Drosophila melanogaster *by Haag-Liautard et al. [26]. Using this mutation rate estimate of 8.4E-9, we date the splits between *S. mimosarum *and *S. tentoriicola *at ~8 million years ago and between *S. lineatus *and *S. mimosarum/S. tentoriicola *at ~13 million years ago (Figure 3). We note that the mutation rates considered here may differ from the actual mutation rate in *Stegodyphus *species and that these time estimates should be considered with caution.

## Conclusions

We identified 1,792 three-way ortholog genes that provide a unique resource for comparative studies in ecology, eco-genomics, population genetics and molecular evolution that relate to the evolution of sociality in the spider genus *Stegodyphus*. Preliminary analyses suggest depleted heterozygosity and relaxed selection in the inbreeding social species *S. mimosarum *relative to outcrossing congeners. However, relaxed selection could not be detected in the substitution pattern, suggesting a recent transition to sociality.

## Materials and methods

Live specimens were collected from Israel (*S. lineatus*) and South Africa (*S. mimosarum *and *S. tentoriicola*) and brought to Aarhus University, Denmark. Total RNA was extracted from the entire body of one individual of each species and complementary DNA (cDNA) was synthesized using Evrogen SMART adapters [27] (Evrogen: http://www.evrogen.com). cDNA libraries were sequenced using the Genome Sequencer FLX instrument (454, Roche) at the Gene Pool facility at the University of Edinburgh. The raw sequence reads and assembled contigs can be downloaded (EBI: ERP001169, Genbank: JT023378 - JT033759 (*S. lineatus*), JT033760 - JT040598 (*S. mimosarum*), JT040599 - JT047773 (*S. tentoriicola*)).

For the assembly of the sequence reads we applied GS De Novo Assembler Software (2.3) using transcriptome assembly default settings. For the *S. mimosarum *assembly an additional -large option for large genomes was used, since the assembly could otherwise not be completed. The assembled contigs (isotigs in GS De Novo Assembler) represent transcripts. Alternative splicing was quantified based on isotig clustering by GS De Novo Assembler software. The isotig clusters (isogroup in GS De Novo Assembler) are referred to as putative genes in the results.

Assembled transcripts were annotated using blastx search against the NCBI non-redundant protein database (http://www.ncbi.nlm.nih.gov) with 10^-6 ^E-value cut-off. Sequences were functionally annotated using the program Blast2GO [28] with default settings. Annotation against *Drosophila *UniProtKB (http://www.uniprot.org) was performed in Bioedit 7.0.4.1 [29]. local blastx with 10^-6 ^E-value cut-off and default settings.

A reciprocal best similarity hit method was used for the ortholog sequence identification, where triplets of reciprocal best hits were selected to represent a putative ortholog set of transcripts. For simplicity they are referred to as ortholog sequences in the results. Nucleotide BLAST was used for the similarity search with default parameters and an E-value cut-off of 10^-5 ^using Bioedit 7.0.4.1 [29]. First, a pair-wise reciprocal blast was performed of which the best hits based on bit scores were selected. If the best hits were similar in both directions, the sequences were scored as putative ortholog between the two species. The putative three-way ortholog sequences were selected from these loci if they were putative orthologs in all three way comparisons. The reciprocal best hit selection was performed using a set of Java scripts implemented in a locally developed sequence manipulation tool (available on request). Putative ortholog sequences between *S. lineatus *and *Acanthoscurria gomesiana *were detected based on the same principle using identified orthologs to query in *S. lineatus*.

For identifying polymorphisms we developed a custom Python script SNP_finder.py (available on request) to mine polymorphic sites from GS De Novo Assembler output files. The polymorphisms were called from sites with a coverage range of 10-150. Minor allele frequency was required to be > 0.3 and only contigs longer than 100 were used, excluding 10 nucleotides from each edge of an assembly. Detected SNPs were assigned ancestral or derived by assuming that the nucleotide found in the same position in the two other species was the ancestral nucleotide. The SNP was assigned non-synonymous if the derived nucleotide changed the amino acid, and synonymous if not.

From the detected ortholog sequences we generated alignments of protein coding regions. The longest open reading frames of detected ortholog sequences, obtained with the online version of Virtual Ribosome [30], were aligned using the alignment program PRANK [31]. The alignments were manually edited assuming that most frame shift errors are caused by sequencing or assembly errors. All sequences were cut after a frame shift gap due to uncertainties of the correct reading frame. All gaps divisible by three were moved to the closest inframe position. To reduce the risk of false positive open reading frames, alignments shorter than 189 bp were discarded. This threshold was set assuming that the number of stop codons in a random sequence with length *n *follows a binomial distribution

(n!/k!(n-k)!)*p∧k*(1-p)∧(n-k)

where *k *is the number of stop codons, and *p *is the probability of the random occurrence of a stop codon, i.e. 3/64. We set the probability to achieve an ORF (*k *= 0) with a length n to 0.05. From this equation the minimum length of ORFs was solved.

(n!/0!(n-0)!)*3/64∧0*(1-3/64)∧(n-0)>0.05∣(n!/0!(n-0)!)=1(since 0!=1and n-0=n),3/64∧0=1

$$\begin{array}{c} 1*1*\left(61/64\right)\wedge \text{n}>0.05|\text{log}\\ \text{n}*\text{log}\left(\text{61}/\text{64}\right)\;>\text{ log}\left(0.0\text{5}\right)\;|:\text{log}\left(\text{61}/\text{64}\right)\\ \text{n }>\text{ log}\left(0.0\text{5}\right)/\text{log}\left(\text{61}/\text{64}\right)\\ \text{n }>\text{ 62}.\text{3991}\\ \text{n }=\;>\text{ 63} \end{array}$$

Thus, to set the probability of a random ORF occurring at below 5%, 63 was selected as ORF length threshold.

The alignments with ORFs (1345 in total) were assigned a random number, and sequences were concatenated in ascending order to form 20 sets of alignments with an equal number of genes. The concatenated genes were used to estimate the transcriptome-wide substitution rates in protein coding genes. The pair-wise proportion of sequence diversity was calculated as the proportion of variable sites from the total. To estimate the rate of synonymous (dS), non-synonymous (dN) and the dN/dS ratio (ω) for each branch of the phylogeny, we used maximum likelihood estimations implemented in the program codeml in the PAML 4.2 package [32]. Codon usage bias of the aligned transcripts was measured as the GC content in silent sites and the effective number of codons was computed using the formula by Novembre [33] calculated with the software INCA 2.1 [34].

## Authors' contributions

JSB, MHS and TB designed the study. TMM and TTH carried out bioinformatics analyses. TMM and JSB carried out the data analyses. TMM, JSB, MHS and TB wrote the paper. All authors read and approved the final manuscript.

## Supplementary Material

Additional file 1 **Procedure for three way orthology identification**. A) Three-way ortholog sequences were selected if they were detected as reciprocal best hits (arrows) in all three two-way comparisons. B) Only one comparison had reciprocal best hits. C) In some cases the reciprocal best hit was detected in two comparisons but not in the third. These were not accepted as three way ortholog sequences. Click here for file

Additional file 2 **PCR primer sequences**. A set of 10 PCR primer sets that amplify in all tested *Stegodyphus *species (*S. lineatus, S. mimosarum, S. tentoriicola, S. dumicola, S. sarasinorum, S. bicolor, S. mirandus, S. tibialis*). The loci amplified are named after the *S. lineatus *transcript name. Click here for file

Additional file 3 **Nucleotide alignments of three way ortholog sequences**. This file contains 1345 alignments of nucleotide sequences found to be ortholog in S. *mimosarum, S. lineatus*, and *S. tentoriicola *with open reading frames. Click here for file

## References

[B1] Lubin Y Bilde T The evolution of sociality in spiders Advances in the Study of Behavior 2007 37 83 145

[B2] Kraus O Kraus M The genus *Stegodyphus *(Arachnida, Araneae). Sibling species, species groups, and parallel origin of social living Verhandlungen des naturwissenschaftlichen Vereins Hamburg 1988 30 151 254

[B3] Johannesen J Lubin Y Smith DR Bilde T Schneider JM The age and evolution of sociality in *Stegodyphus *spiders: a molecular phylogenetic perspective Proceedings of the Royal Society B-Biological Sciences 2007 274 231 237 10.1098/rspb.2006.3699 PMC1685853 17148252

[B4] Charlesworth D Effects of inbreeding on the genetic diversity of populations Philosophical Transactions of the Royal Society of London Series B-Biological Sciences 2003 358 1051 1070 10.1098/rstb.2003.1296 PMC1693193 12831472

[B5] Charlesworth D Willis J The genetics of inbreeding depression Nature Reviews Genetics 2009 10 783 796 10.1038/nrg2664 19834483

[B6] Vera JC Wheat CW Fescemyer HW Frilander MJ Crawford DL Hanski I Marden JH Rapid transcriptome characterization for a non-model organism using 454 pyrosequencing Molecular Ecology 2008 17 1636 1647 10.1111/j.1365-294X.2008.03666.x 18266620

[B7] Mayer KFX Taudien S Martis M Simkova H Suchankova P Gundlach H Wicker T Petzold A Felder M Steuernagel B Scholz U Graner A Platzer M Dolezel J Stein N Gene content and virtual gene order of barley chromosome 1H Plant Physiology 2009 151 496 505 10.1104/pp.109.142612 19692534 PMC2754631

[B8] O'Neil ST Dzurisin JDK Carmichael RD Lobo NF Emrich SJ Hellmann JJ Population-level transcriptome sequencing of non-model organisms *Erynnis propertius *and *Papilio zelicaon* BMC Genomics 2010 11 310 10.1186/1471-2164-11-310 20478048 PMC2887415

[B9] Shulaev V The genome of woodland strawberry (*Fragaria vesca*) Nature Genetics 2011 43 109 116 10.1038/ng.740 21186353 PMC3326587

[B10] Clark MS Thorne MAS Toullec J-Y Meng Y Guan LL Peck LS Moore S Antarctic Krill 454 Pyrosequencing Reveals Chaperone and Stress Transcriptome PLoS ONE 2011 6 e15919 10.1371/journal.pone.0015919 21253607 PMC3017093

[B11] Li R The sequencing and de novo assembly of the giant panda genome Nature 2010 463 311 317 10.1038/nature08696 20010809 PMC3951497

[B12] Vicario S Moriyama EN Powell JR Codon usage in twelve species of *Drosophila* BMC Evolutionary Biology 2007 7 226 10.1186/1471-2148-7-226 18005411 PMC2213667

[B13] Jorgensen F Schierup MH Clark AG Heterogeneity in regional GC content and differential usage of codons and amino acids in GC-poor and GC-rich regions of the genome of *Apis mellifera* Molecular Biology and Evolution 2009 24 611 619 17150976 10.1093/molbev/msl190

[B14] Cutter AD Wasmuth JD Blaxter ML The Evolution of Biased Codon and Amino Acid Usage in Nematode Genomes Mol Biol Evol 2006 23 2303 2315 10.1093/molbev/msl097 16936139

[B15] Heger A Pontig CP Evolutionary rate analyses of orthologs and paralogs from 12 *Drosophila *genomes Genome Research 2007 17 1837 1849 10.1101/gr.6249707 17989258 PMC2099592

[B16] Zhang LQ Li WH Mammalian housekeeping genes evolve more slowly than tissue-specific genes Molecular Biology and Evolution 2004 21 236 239 14595094 10.1093/molbev/msh010

[B17] Johannesen J Wickler W Seibt U Moritz RFA Population history in social spiders repeated: colony structure and lineage evolution in *Stegodyphus mimosarum *(*Eresidae*) Molecular Ecology 2009 18 2812 2818 10.1111/j.1365-294X.2009.04238.x 19500247

[B18] Agnarsson I Maddison WP Avilés L Complete separation among matrilines in a social spider metapopulation inferred from hypervariable mitochondrial DNA region Molecular Ecology 2010 19 3052 3063 10.1111/j.1365-294X.2010.04681.x 20598078

[B19] Charlesworth D Wright SI Breeding system and genome evolution Current Opinions in Genetics & Development 2001 11 685 690 10.1016/S0959-437X(00)00254-9 11682314

[B20] Lynch M Conery J Burger R Mutation accumulation and the extinction of small populations American Naturalist 1995 146 489 518 10.1086/285812

[B21] Forman M Kral J Haddad CR The cytogenetic approach reveals speciation events in social spiders *Stegodyphus *(Araneae: Eresidae) Book of Abstracts, 18th International Congress of Arachnology 2010, Siedlce, Poland

[B22] Miller JA Carmichael A Ramirez MJ Spagna JC Haddad CR Rezac M Johannesen J Kral J Wang XP Griswold C Phylogeny of entelegyne spiders: affinities of the family *Penestomidae *(NEW RANK), generic phylogeny of *Eresidae*, and asymmetric rates of change in spinning organ evolution (*Araneae, Araneoidea, Entelegynae*) Molecular Phylogenetics and Evolution 2010 55 786 804 10.1016/j.ympev.2010.02.021 20206276

[B23] Lorenzini DM da Silva PI Jr Soares MVB Arruda P Setubal J Daffre S Discovery of immune-related genes expressed in hemocytes of the tarantula spider Acanthoscurria gomesiana. Developmental and Comparative Immunology 2006 30 545 556 10.1016/j.dci.2005.09.001 16386302

[B24] Selden PA Gall JC A Triassic mygalomorph spider from the northern Vosges, France Palaeontology 1992 35 211 235

[B25] Selden PA Anderson JM Anderson HM Fraser NC Fossil araneomorph spiders from the Triassic of South Africa and Virginia The Journal of Arachnology 1999 27 401 414

[B26] Haag-Liautard C Dorris M Maside X Macaskill S Halligan DL Charlesworth B Keightley PD Direct estimation of per nucleotide and genomic deleterious mutation rates in *Drosophila* Nature 2007 445 82 85 10.1038/nature05388 17203060

[B27] Zhu YY Machleder EM Chenchik A Li R Siebert PD Reverse transcriptase template switching, a SMART approach for full-length cDNA library construction Biotechniques 2001 30 892 897 11314272 10.2144/01304pf02

[B28] Conesa A Götz S Garcia-Gómez J M Terol J Talón M Robles M Blast2GO: A universal tool for annotation, visualization and analysis in functional genomics research Bioinformatics 2005 21 3674 3676 10.1093/bioinformatics/bti610 16081474

[B29] Hall TA BioEdit: a user-friendly biological sequence alignment editor and analysis program for Windows 95/98/NT Nucleic Acids Symposium Series 1999 41 95 98

[B30] Wernersson R Virtual Ribosome - a comprehensive DNA translation tool with support for integration of sequence feature annotation Nucleic Acids Research 2006 34 w385 w388 10.1093/nar/gkl252 16845033 PMC1538826

[B31] Löytynoja A Goldman N Phylogeny-aware gap placement prevents errors in sequence alignment and evolutionary analysis Science 2008 320 1632 1635 10.1126/science.1158395 18566285

[B32] Yang Z PAML 4: Phylogenetic analysis by maximum likelihood Molecular Biology and Evolution 2007 24 1586 1591 10.1093/molbev/msm088 17483113

[B33] Novembre JA Accounting for background nucleotide composition when measuring codon usage bias Molecular Biology and Evolution 2002 19 1390 1394 10.1093/oxfordjournals.molbev.a004201 12140252

[B34] Supek F Vlahovicek K INCA: synonymous codon usage analysis and clustering by means of self-organizing map Bioinformatics 2004 20 2329 2330 10.1093/bioinformatics/bth238 15059815

