# Supplementary figures and images for: Orthologous genes identified by transcriptome sequencing in the spider genus Stegodyphus
Source: BMC Genomics. 2012 Feb 14;13:70. doi: 10.1186/1471-2164-13-70 (PMC3350440; doi:10.1186/1471-2164-13-70)

A.

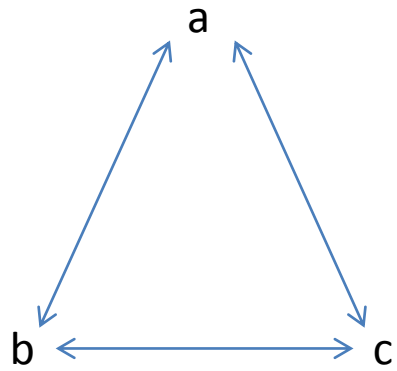

B.

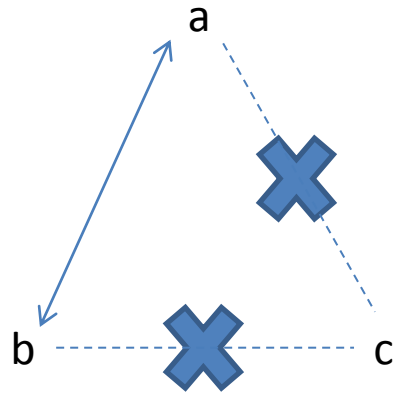

C.

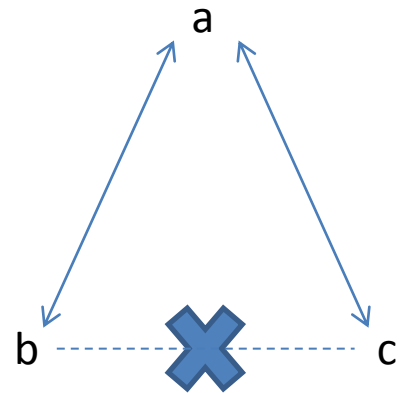

Supplement: Additional file 1 — Procedure for three way orthology identification. A) Three-way ortholog sequences were selected if they were detected as reciprocal best hits (arrows) in all three two-way comparisons. B) Only one comparison had reciprocal best hits. C) In some cases the reciprocal best hit was detected in two comparisons but not in the third. These were not accepted as three way ortholog sequences. [file 1471-2164-13-70-S1.PDF]
