# Supplementary material for: Orthologous genes identified by transcriptome sequencing in the spider genus Stegodyphus
Source: BMC Genomics. 2012 Feb 14;13:70. doi: 10.1186/1471-2164-13-70 (PMC3350440; doi:10.1186/1471-2164-13-70)
Supplement: Additional file 2 — PCR primer sequences. A set of 10 PCR primer sets that amplify in all tested Stegodyphus species (S. lineatus, S. mimosarum, S. tentoriicola, S. dumicola, S. sarasinorum, S. bicolor, S. mirandus, S. tibialis). The loci amplified are named after the S. lineatus transcript name. [file 1471-2164-13-70-S2.DOC]

**Additional file 2**

**lineatusisotig04264**

CAGTWGAGTTAGAACATCCTCG – RTCGTGAAACWGTRAGGC (Annealing temperature: 51 degrees)

**lineatusisotig01117**

GTCTGAGCAAYAACCGYGGC – GTTCCTCTATCAATGCCAGRG (Annealing temperature: 53 degrees)

**lineatusisotig02215**

GYTTCATACCTCCTGATGGTG – CATACAAKCCACTTCTTCC (Annealing temperature: 51 degrees) **lineatusisotig02572**

GAAGGCAATGGAAGGTGGACG – CCAAAATACAATAACTGCTCTG (Annealing temperature: 53 degrees)

**lineatuscontig00126**

ACTGCTGCTCTTGGTCTCC – GACTGAGCAATCRTRTCCAGAG (Annealing temperature: 53 degrees)

**lineatusisotig02165**

GTACTGCATCTTCTGGYCAG – TATACACTGGACCATAGGATCTG (Annealing temperature: 53 degrees)

**lineatusisotig02166**

GCTATGGATGGTACTGAAGGC – CCTGGWGGCTCATTCATCTGTCC (Annealing temperature: 53 degrees)

**lineatusisotig01622**

CTGAAGAGCATAGGAACGGC – CCGTCWTCTGTCTGTSTAGTTGG (Annealing temperature: 53 degrees)

**lineatusisotig01837**

GTCCAAGCHAAAGCACAGG – TAGCTGCATTCTGAACCTGAG (Annealing temperature: 53 degrees)

**lineatusisotig05431**

TGGCCTTTCCAYCAGTC – GKACATTGATATTCACCTTCAG (Annealing temperature: 50 degrees)
